# Healthy and Happy? An Ethical Investigation of Emotion Recognition and Regulation Technologies (ERR) within Ambient Assisted Living (AAL)

**DOI:** 10.1007/s11948-024-00470-8

**Published:** 2024-01-25

**Authors:** Kris Vera Hartmann, Giovanni Rubeis, Nadia Primc

**Affiliations:** 1 Institute for the Study of Christian Social Service (DWI), Theological Faculty, Heidelberg University, Karlstr.16, 69117 Heidelberg, Germany; 2 Division Biomedical and Public Health Ethics, Karl Landsteiner University of Health Sciences, Dr.-Karl-Dorrek-Str. 30, Krems, 3500 Austria; 3 Institute of History and Ethics of Medicine, Medical Faculty, Heidelberg University, Im Neuenheimer Feld 327, 69120 Heidelberg, Germany

**Keywords:** Ambient assisted living (AAL), Emotion recognition, Emotion regulation, Ethics, Privacy, Autonomy

## Abstract

Ambient Assisted Living (AAL) refers to technologies that track daily activities of persons in need of care to enhance their autonomy and minimise their need for assistance. New technological developments show an increasing effort to integrate automated emotion recognition and regulation (ERR) into AAL systems. These technologies aim to recognise emotions via different sensors and, eventually, to regulate emotions defined as “negative” via different forms of intervention. Although these technologies are already implemented in other areas, AAL stands out by its tendency to enable an inconspicuous 24-hour surveillance in the private living space of users who rely on the technology to maintain a certain degree of independence in their daily activities. The combination of both technologies represents a new dimension of emotion recognition in a potentially vulnerable group of users. Our paper aims to provide an ethical contextualisation of the novel combination of both technologies. We discuss different concepts of emotions, namely Basic Emotion Theory (BET) and the Circumplex Model of Affect (CMA), that form the basis of ERR and provide an overview over the current technological developments in AAL. We highlight four ethical issues that specifically arise in the context of ERR in AAL systems, namely concerns regarding (1) the reductionist view of emotions, (2) solutionism as an underlying assumption of these technologies, (3) the privacy and autonomy of users and their emotions, (4) the tendency of machine learning techniques to normalise and generalise human behaviour and emotional reactions.

## Introduction

An increasing number of technologies try to capture human emotions in different domains of society as part of e.g. security systems, education, work place surveillance, or detection of psychiatric diseases (Crawford, 2021, p. 151; Boyd & Andalibi, 2023). Ambient Assisted Living (AAL) is a specific domain in health care that seems predestined for the implementation of emotion recognition devices, as they are already fitted with numerous monitoring sensors. AAL systems encompass technologies that track the daily activities of different groups of users (e.g. older people and people with disabilities or chronic diseases) in their private living space to enhance their autonomy and reduce their need for informal and/or professional care (Manzeschke et al., 2016; Offermann-van Heek et al., 2019; Sapci & Sapci, 2019; Queirós et al., 2017; Rubeis, 2020; Sixsmith, 2013). The newest generation of AAL systems use machine learning and Big Data approaches to continuously collect and process large amounts of user data from their most private everyday activities.

Recent technological developments show that there are increasing efforts to further extend the continuous tracking of the daily lives of users and to influence their behaviour by integrating automated emotion recognition and regulation (ERR) into AAL systems. The combination of both technologies aims to identify the users’ emotions via different sensors and to regulate emotions that are defined as “negative”. When the system detects a negative emotion, it notifies caregivers or users themselves, or launches an automated intervention, such as suggesting an activity, or changing the music or light scheme.

Currently, these technologies still play a rather minor role in the technological development of AAL systems. Their ethical status, in contrast, is all the more debatable. The combination of both technologies can be seen as an attempt to extend the daily and continuous tracking of everyday activities to the emotional states of a group of users that rely on these assistive technologies to maintain a certain degree of autonomy. People do not necessarily want to share their emotions openly with others, especially not with non-related third parties such as professional caregivers or doctors. The right to decide with whom and to what extent we want to share our emotions can be regarded as part of our right to “emotional privacy“ (Roemmich et al., 2023).

The combination of AAL and ERR stands out from other forms of emotion recognition as AAL offers an inconspicuous 24-hour surveillance in the private living space of users who rely on the technology to maintain a certain degree of independence in their daily activities. This represents a new dimension of emotion recognition in a potentially vulnerable group of users. Our paper aims to provide an ethical contextualisation of the novel combination of both technologies, which, to the best of our knowledge, is still missing in the general discussion of AAL and ERR technologies.

Crawford (2021) critically investigated the historical origins, scientific concepts, as well as the ethical implications of the broader field of emotion recognition in facial recognition technologies. Stark and Hoey (2021) focus on current AI-based emotion recognition systems in general and provide a taxonomy of conceptual models and proxy data used. Mohammad (2022) highlights the reductionistic stance of ERR systems and discusses the fundamental rights (e.g. right to privacy, freedom of expression, right to protest) which can be affected by the use of these technologies. In our paper, we go beyond these fundamental approaches and discuss ethical issues that result from an uncritical implementation of reductionist emotion theories by technology developers in the field of AAL. We address specific ethical challenges that are related to the overall intention of implementing automated ERR in AAL.

After a description of our method (section “Method”) we present and discuss the various types of ERR technologies that are currently developed for the specific use in AAL systems and the different models of emotions that form the basis of these technologies (section “ERR in AAL-Systems: Types of Technological Solutions and their Respective Challenges”). We then highlight four ethical questions that arise in the context of ERR in AAL systems (section “Ethics of Emotion Recognition in AAL”).

## Method

Our analysis is part of a larger research project that has focused on the question of whether and how fundamental and widely discussed ethical aspects of AAL technologies are reflected in the current technological development of AAL. The research databases PubMed, Science-direct, and Web of Science were used to compile the sample of research papers that form the basis of the present inquiry. Manual searches were performed for the timespan from 2004 (when the first article with the keyword AAL is listed in the database PubMed) till 2023 (reference date: 10/31/23), using the search terms “Ambient Assisted Living“ and ”AAL“ in combination with ”emotion recognition”. After initial screening of the titles and abstracts, duplicates and papers with other application areas were removed. 30 papers were included in the further screening process. Inclusion criteria were original research papers in the field of AAL describing the technological development of systems that attempt to detect the users’ emotions with sensors and optionally offer interventions with a focus on regulating emotions. We excluded reviews, as well as publications that focus only the detection of stress (vs. no stress) without differentiating between different emotional states. 15 papers were included in the final sample (see Table 1). We performed a qualitative content analysis to identify different technologies and concepts used in ERR. For the ethical investigation, we used a narrative synthesis to relate the qualitative findings to relevant research from the fields of philosophy, medical ethics, sociology, and critical AI research that deal with emotions, ERR, and AAL, and that formed the framework of our overarching research project. We discuss the results of the ethical analysis and identify the four most important aspects.

**Table 1:** Compilation of the research sample

Compilation of the research sample	
1. Manual Search (PubMed, Science Direct, Web of Science), search terms: “Ambient assisted living” OR “AAL” AND “Emotion recognition” (Inclusion criteria: research papers from computer science OR engineering science)	187
2. Title and abstract screening: Exclusion of thematically not relevant papers, duplicates, other forms of publication than original research papers	-157
3. Full text screening: Exclusion of articles not matching the definition of emotion recognition technology	-15
**Included articles**	**15**

## ERR in AAL-Systems: Types of Technological Solutions and Their Respective Challenges

The relatively small number of papers included in the final compilation is itself an important result of our research, as it shows that developers working in the field of AAL only begin to gradually include ERR technologies into the flourishing field of AI-based AAL systems. Table 2 provides an overview of the different technologies discussed in the papers of our research sample, including sensor-types, proxy data, the type of regulation, and the emotion model that authors refer to as a basis of their technology. If no explicit emotion model is mentioned by the authors, we provide information concerning the categories used to identify different emotions and their possible connection to established emotion models. As the table shows, most papers use either the Basic Emotion Theory (BET) by Ekman & Friesen (1971), Ekman et al. (1987, 2002), or the Circumplex Model of Affect (CMA), introduced by Russell (1980), (or both) as a basis of their research.

1987

**Table 2:** Overview of emotion recognition technologies in the research sample

	Sensors/Devices	Proxy data	Regulation	Emotion Concept
Griol et al. (2014)	Conversational/Natural language interface	Acoustic features and dialog information derived from speech	-	No explicit emotion model mentioned, preliminary work (Callejas and López-Cózar, 2008) mentions CMA and Scherer, 2005
Matiko et al. (2015)	Wearable EEG headband	EEG signals	-	No explicit emotion model mentioned, identification of low vs. high valence (i.e. simplified form of CMA)
Mano et al. (2016)	Cameras	Facial expressions	Information of caregivers	BET, CMA
Meza-Kubo et al. (2016)	EMOTIV EPOC + Headset (neuroheadset)	EEG signals	-	No explicit emotion model mentioned, differentiation between pleasant and unpleasant emotions
Lozano‑Monasor et al. (2017)	Webcamera	Facial expressions	-	BET
Yaddaden et al. (2018)	Cameras, radio frequency identification (RFID)-tags on objects	Facial expressions, radio frequency	-	BET
Rus et al. (2018)	Couch with capacitive proximity sensors	Electric field caused by presence and motion	Light	BET, CMA
Rincon et al. (2019)	Robot EmIR	Facial expression	Simulation of empathetic behavior by robot	No explicit emotion model mentioned, use of KDEF database (Calvo & Lundqvist, 2008) that explicitly builds on BET
**Al Machot (2019)**	Electrodermal	Electrodermal activity (EDA)	-	CMA
Costa et al. (2019)	Wristband with different sensors	Galvanic skin response (GSR), temperature, photoplethysmography, accelerometer, gyroscope	Rescheduling activities and events; information of caregivers	CMA
Nie et al. (2021)	Glasses-like device with cameras	Facial expressions, eye and eyebrow movements, head movement, pupillometry	Display alarms on desktop or smartphone app	BET, CMA
Calatrava-Nicolás et al. (2021)	Ambient sensors, wearable	Accelerometry, skin temperature, electrodermal activity, blood volume pulse	Display of emotional coaching strategies, information of caregivers	CMA
Elkobais and Al Machot (2021**)**	Cameras, IoT	Facial expressions, modeling of situational aspects by end users via IoT	-	BET, CMA, as well as a set of user-defined emotions
Babli et al. (2021)	Wristbands, robot with camera	Volumetric variations of blood circulation (PPG sensor) for detection of stress, facial expressions for identification of emotions	Robot suggests activities	BET

### Ethical Justification of ERR in AAL

On a normative level, the development and implementation of AAL systems is generally justified on the basis of health-related objectives such as the goal to minimise the need of assistance, or to enhance the well-being of persons in need of care. Since this involves the automated collection of very private and personal data, one of the key ethical questions is whether and why AAL systems should be expanded by automated recognition and regulation of emotions. Two lines of argumentation can be identified in the papers in our sample. Several authors argue that ERR will help to enhance the well-being or quality of life of users (e.g. Mano et al., 2016; Calatrava-Nicolás et al., 2021; Nie et al., 2021). Positive emotions are, for example, regarded as crucial for recovering from a disease (Mano et al., 2016), or EER as helpful for an early detection of mental health issues (Nie et al., 2021). The second line of argumentation justifies the implementation of ERR as a way to generally improve the human-machine interaction and technical assistance provided by AAL (Rus et al., 2018; Yaddaden et al., 2018; Al Machot et al., 2019; Costa et al., 2019, Rincon et al., 2019). For both lines of justification, the question arises, whether the developed technologies are actually suitable for achieving these goals. As we will argue below, this seems rather questionable given the reductionist view of emotions that form the basis of these technological attempts at ERR.

### The Reinterpretation and Implementation of BET and CMA in AAL Technology

Most papers in our sample either explicitly or implicitly rely on BET or CMA (or both) as a basis for their technological development. However, none of these theories was originally designed for automatic ERR. As automatic ERR measures proxy data, not emotions themselves (Stark & Hoey, 2021), the research groups had to simplify and mathematise both approaches with the goal to use physiological and bodily signs as proxy data for emotions. Only two papers use additional information, namely dialog information that is extracted via a natural language interface (Griol et al., 2014), and contextual information that is collected by Internet of Things (IoT) applications and can be individually linked by the end users to a set of user-defined emotions, such as “watching TV on Sunday” and “happy” (Elkobais & Al Machot, 2021). These proxy data are used to identify the emotions that a person is supposed to be currently experiencing. Table 2 gives an overview of the proxy data that are used in the papers of our research sample. However, physiological processes are just one aspect of the complex phenomenon of emotions. Picard, whose work has been a milestone in linking emotions to information technology, emphasizes that although expressive patterns of emotions are influenced by socio-cultural factors, as well as gender, personality, and temperament (Picard, 2000), automated ERR is largely limited to the tracking and modelling of physiological aspects. Especially facial expressions and their interpretation form the basis of the technology, for which the BET by Ekman and colleagues is a crucial tool (Crawford, 2021).

In their studies in the 1960s and 1970s, psychologist Paul Ekman and his colleagues (Ekman & Friesen, 1971) explored the thesis of the universality of certain emotions, thus their independence from cultural patterns of expression and interpretation. In a famous experiment, members from an indigenous community in New Guinea, who had little contact with Western culture, were asked to match photographs of facial expressions with narrated stories. Ekman and his collaborators claimed that their experiment confirmed their previously established thesis of six basic and universal emotions (happiness, anger, fear, disgust, sadness, and surprise), despite some difficulties in distinguishing between fear, surprise, anger and disgust. In the last decades the BET, especially the underlying experimental design and thesis of the universality of emotions were questioned by several researchers (e.g. Fridlund, 1994; Russell, 1994; Barrett et al., 2019).

Furthermore, the implementation and validity of the BET in affective computing has been the specific object of criticism, as for example the same facial expression (e.g. a smile) may be related to different emotional states in a specific person (Kappas, 2010), a variability that may be even greater between several individuals. Except for Elkobais and Al Machot (2021), who try to individualise their approach by integrating contextual information about daily activities that can be linked by the end-user themselves to a set of user-defined emotions, these challenges are not explicitly discussed or reflected upon by the authors of the papers in our sample. Instead the authors uncritically assume the validity of the basic approach, i.e. the intra- and interpersonal validity of the technological inference of emotional states from facial expressions. They hereby ignore that Ekman et al. themselves rejected their underlying assumption of a universal nexus between facial expressions and underlying emotions in their later work. Ekman and colleagues acknowledged that there is a stronger intercultural and interpersonal variability in that nexus (Ekman et al., 1987) than they initially postulated as well as the importance of additional features (e.g. concurrent speech, body movements) in the identification and expression of emotions (Ekman et al., 1987). Both aspects undermine the intention of automated ERR using facial expressions.

As already mentioned, these challenges are in the vast majority of cases not reflected or addressed in the technological publications in our sample. Mano and colleagues (2016), for example, use the basic emotions of BET and relate them without further reflection to measurable facial expressions. The authors interpret basic emotions quite uncritically as “innate and culturally uniform”. All other emotional categories are then built up from combinations of these basic emotions” (Mano et al., 2016, p. 183). Rus and colleagues (2018), who refer to both BET and CMA, use an even more reductionist account. To protect the privacy of users and to ensure their non-identifiability, they decide not to use facial expressions, but body posture and movement with the help of proximity sensors built in a couch (Rus et al., 2018).

**Fig. 1:**
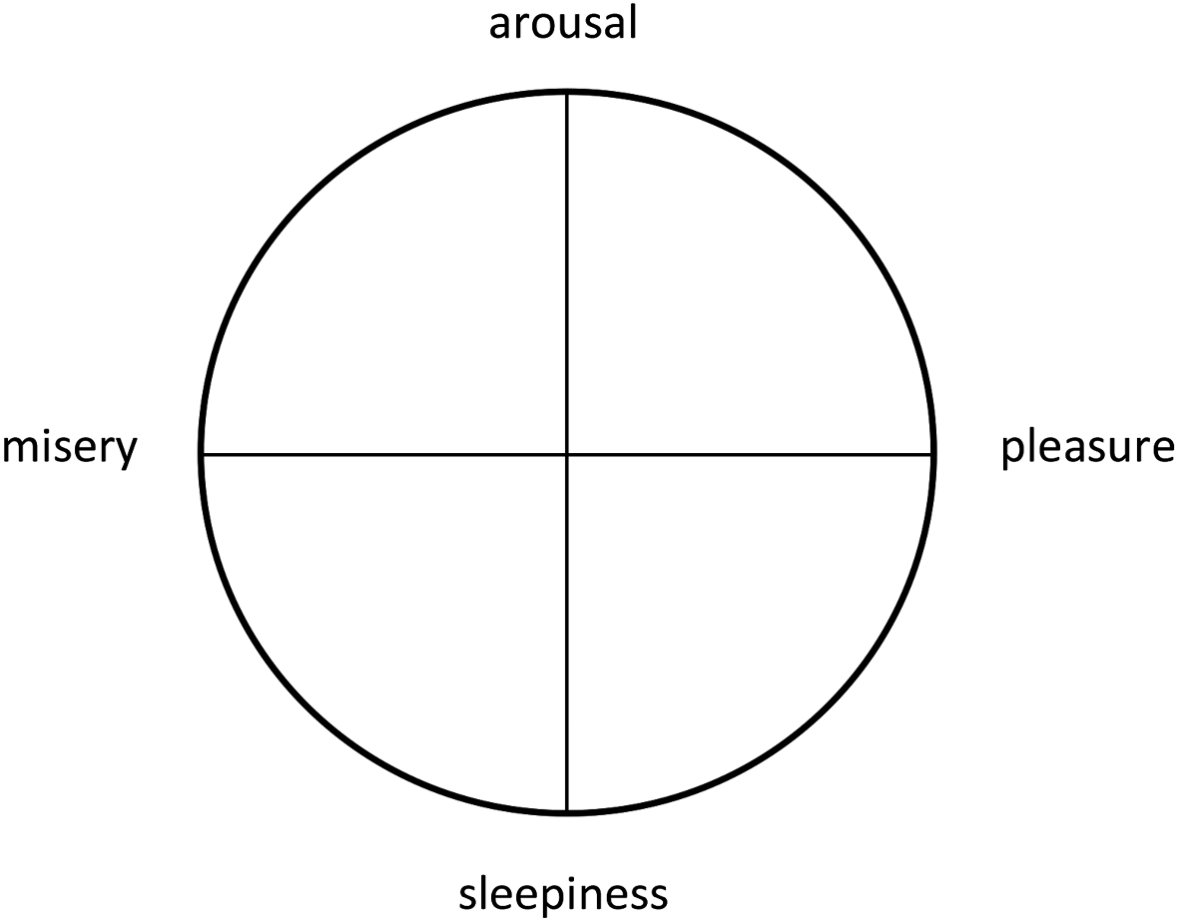
Schematic illustration of the Russel’s circumplex model (Russell, 1980), own illustration

The more complex CMA was introduced by Russell (1980), a critic of BET who engaged in ongoing scientific debates with Ekman (Russell, 1994; Ekman 1995; Russell, 1995; Russell et al., 2011). Russell developed a circular model (Fig. 1) divided by two axes (more or less pleasure on the horizontal line; more or less arousal on the vertical line) which allows to identify and to relate different affective states to one another (see Fig. 1). The two-dimensional model was chosen because Russell believed that it is a good “representation of the cognitive structure that laymen utilize in conceptualizing affect.” (Russell, 1980, p. 1161). The CMA was never intended as a tool to correctly identify emotions that people currently experience, but rather as an “implicit taxonomy” (Russell, 1980, p. 1162) which helps people organize their knowledge on emotions. Russell intended this implicit taxonomy or cognitive structure to help “interpreting verbal descriptions of emotion, including anything from a subtle hint to an explicit declaration.” (Russell, 1980, p. 1162).

The vast majority of the technological studies in our sample (except for Griol et al., 2014, who include dialog information via a natural language interface) ignore the explicit linguistic anchoring of the CMA. Theyimplicitly reinterpret it in the context of AAL, stating that emotions can apparently be technically measured and identified with recurrence to physiological data instead of linguistic expressions. The authors neither critically question nor justify whether and how such a reinterpretation may lead to valid and reliable inferences. Al Machot and colleagues (2019) implicitly reinterpret the linguistic features of CMA as a representation of the different psychological dimensions of emotions as lived experiences (Al Machot et al., 2019). Based on their reinterpretation of CMA, they develop a model that tries to capture two variables (valence and arousal) in order to map different emotions. For example, a high valence and a high arousal score will be classified as “happy” and “excited”; the combination of low valence and high arousal refers to “anger”, “fear” and “distress”; low valence paired with low arousal refers to “sadness” and “depression”.

As these brief explanations show, most commentators generally adopt BET and CMA quite uncritically and reinterpret them without further reflection in the context of AAL. This not only calls into question the overall validity of the undertaking, but also the ethical justification for implementing these technologies in the context of AAL that, as a health care technology, aims to support people in need of assistance.

### The Distinction Between Fake and Real Emotions

Another shortcoming that results from the uncritical simplification and reinterpretation of BET and CMA in AAL relates to the challenge of correctly distinguishing via technological means between ”real” and “fake” emotions (e.g. happy facial expression without a matching “happy” emotional state). In our sample, only Rus et al. (2018) and Nie et al. (2021) explicitly address this challenge. To not get fooled by “acted” emotions, Rus et al. employ proximity sensors in a couch. Nie et al. rely on bio-signals as they recognize that “a user can easily fake a smile even if s/he is depressed” (Nie et al., 2021, p. 3), whereas bio-signals are not so easily controllable by users and, hence, are supposed to allow more reliable inferences. Although Nie et al. also track facial expressions, they consider them as representing “apparent emotions” in contrast to “real emotions” that are to be identified by measuring four types of bio-signals. Costa et al. (2019) implicitly address the issue of “fake” emotions, by stating the impact of the larger social group on emotions. They claim that people in care institutions are “natural[ly] forming groups with leaders and followers” (Costa et al., 2019, p.481) and they assume that followers subordinate their emotions to those of the leader. Nevertheless, the system of Costa et al. is designed to determine the “real” emotions of group participants by statistically comparing the measured data of the individuals.

### Variability of Emotions

A further challenge for the reliability of automatic ERR in AAL arises from the individual variance in what people feel and how they express their emotions. It is nowadays generally recognised that emotions and their expression are not universal, but individually different, prompting the authors of the analysed papers to consider different solutions. Some try to reach the goal of “subject independence” (Al Machot et al., 2019, p. 3) by using Deep Neural Networks (DNN). Nie et al. (2021) try to generate more reliable and valid results by measuring several variables instead of just one. Costa et al. (2019), on the other hand, approach the problem of variability by implicitly abandoning the universality thesis in favour of an individualistic approach. In order to achieve accurate results, the system will have to be adapted to the particular user and their individual emotional states and reactions. In any case, the issues of individual variability and fake emotions represent a major challenge for the reliability of automatic ERR. Further interdisciplinary research is necessary if this technology is supposed to reliably identify and regulate the daily lived emotions of AAL users.

### Regulation and Forms of Intervention

Even if one were to assume that the above-mentioned challenges will be solved and automated emotion recognition in AAL will eventually be successful and reliable– which at the moment is rather questionable– an additional question emerges: *What to do with the measured emotions in the context of AAL?* Seven papers envision an intervention, which in general aims to regulate the measured emotions towards positive ones. Rus et al. built a system where “[b]ased on emotional state the lightning of the room is adjusted. Invisible sensors in the couch sense the emotional movements and communicate with the lamp” (Rus et al., 2018, p. 264). Mano et al. monitor a user’s daily activities and emotions via cameras and try to construct a normal day for each person. If „signs of unexpected behaviour” are detected, the system “acts on behalf of the patient”, which means that a third person (relative, nurse) is informed about the unexpected behaviour, but “other kinds of actions, as well as different actions for new situations or issues detected and issuing warnings through smartphone notifications, SMS or phone calls” (Mano et al., 2016, p. 181) can be added to the settings. Rincon et al. (2019) use the identification of facial expressions in end users to simulate empathetic behaviour in an interacting robot. Costa et al. (2019) want to determine the emotions of social groups in nursing homes with the goal to schedule and reschedule activities and events with as little interaction as possible (interaction is considered intrusive).

The system of Nie et al. (2021) makes the user wear a glasses-like device that measures facial expressions, eye and eyebrow movements, head movements, and pupillometry. The system notifies the user on a smartphone or desktop app about anomalous values. Calatrava-Nicolás et al. (2021) also use interventions in the form of communication with the user. Here, a vacuum cleaner-sized robot suggests emotional coaching strategies (e.g. to leave the house or meet other people) or informs caregivers when a low mood or “strange behaviour” (such as staying in bed too long) is detected. Babli et al. (2021) also use a robot that suggests activities (reading a book, playing music, offering a glass of water) if negative emotions are detected. Whether these interventions are actually suitable for achieving the general goals of ERR in AAL (see section “Reductionist Emotion Concept”) is something that remains to be explored in further studies and cannot be taken for granted. The change of light or music schemes can certainly be regarded as a rather harmless (and probably not very effective) intervention, which, however, calls into question the ethical justification for collecting corresponding “emotional” data. AAL systems are not designed as lifestyle products that one may decide to use or not to use. Rather, people in need of assistance rely on them to maintain a certain degree of autonomy in their daily activities for as long as possible. Furthermore, it must be noted that (as the example of Mano et al., 2016 shows) the type of intervention (e.g. a rather harmless change of light and music schemes) can in principle be switched at any time to a more invasive one. Even an intervention in the form of a simple notification of a caregiver involves a risk of pathologising any unusual deviation in the physiological patters of users (an issue that will be further discussed under section “Human Variability and Machine Normativity”) and does not necessarily enhance as intended the well-being or autonomy of users.

Against the background of all these challenges, a number of ethical questions arise, which we will address in the next section.

## Ethics of Emotion Recognition in AAL

Based on a narrative synthesis of relevant research from the fields of philosophy, medical ethics, sociology, critical AI studies, and AAL, we highlight two groups of ethical issues. The first group refers to the underlying conceptual assumptions of both technologies (AAL and ERR), namely the (1) reductionist view of emotions as well as (2) solutionism. The second group of ethical issues more specifically addresses the intentional goal of the technologies, as these affect (3) the emotional privacy and autonomy of users as well as (4) the tendency of machine learning techniques to normalise human behaviour.

### Reductionist Emotion Concepts

The examined technological approaches in our sample use rather reductionist conceptualisations and models of emotions which put their reliability and validity in doubt. We have already argued above that BET and CMA are adopted and reinterpreted quite uncritically in the context of AAL. The technological approaches in AAL view emotions as something that can be reduced to or at least be readily identified by a set of physiological parameters. Only two papers include at least some additional information to identify the emotional states of AAL users (Griol et al., 2014; Elkobais & Al Machot, 2021). This ignores the widely recognised psychological, cultural, and social dimension of emotions (Kappas, 2010). The “physical display of emotion is only one facet of emotion.” (McStay & Pavliscak, 2019). Therefore, it can never capture the “full emotional experience of the person” (Mohammad, 2022, pp. 4, 9), as critics of automated ERR stress. The limited validity of these theoretical approaches questions the ethical justification for the automated collection of corresponding data.

Several theoretical and technological approaches have been developed that offer a multi-dimensional and, hence, more complex view of emotions and automated ERR (Lively & Heise, 2014; Fontaine et al., 2007). Some focus on people with dementia (a main target group of AAL technologies) and a possible implementation in AAL technologies (Francis et al., 2020; Robillard & Hoey, 2018). Whether these approaches enable a more reliable automated ERR in the context of AAL that can be ultimately justified with reference to the overall goals of an enhancement of the well-being and autonomy of persons in need of assistance, is beyond the scope of the present paper. It certainly underlines the necessity of an interdisciplinary and ethically reflected development of assistive technologies in the field of AAL (Robillard & Hoey, 2018). Apart from issues of reliability, the more complex approaches are still confronted with the ethical challenges discussed below.

### Solutionism

A constant tenet that can be seen across all approaches is a tendency towards solutionism, which signifies the view that technical fixes can be applied to genuinely social or political problems (Howard, 2021; Morozov, 2013). As an answer to the question, why these technologies should be developed and implemented, solutionism fails due to the misleading framing of the basic problem at hand. Catering to the needs of older or chronically ill adults in order to improve or sustain their quality of life and enable them to live a mostly independent life is primarily a social task. It goes beyond a mere healthcare issue and intersects with issues of health equity, autonomy, person-centeredness, and fundamental concepts of a good life. To define the emotional well-being of older adults and other people in need of care as a problem for which a technical fix can be developed narrows this complex phenomenon down to aspects of manageability, controllability, and effectiveness. Positive emotions are defined as a goal that is to be achieved by technical means. This ignores the aforementioned complexity and individuality of emotions (see section “Reductionist Emotion Concepts”). An over-simplistic stimulus-reaction-model is applied, which, ignores the far richer and more complex concepts of emotions in research. This undermines the very goals of AAL-technology, mainly catering to the individual needs of their users, by treating emotions as a standardisable issue for which a standardised technological solution can be provided. Narrowing down this complex issue in terms of a solutionist approach might also obscure alternatives to technology use in this respect, above all the improvement of intersectoral health care as well as social services. It is highly questionable whether ERR technologies are the right approach to ensure quality of life of their users and create personalised health services.

### Issues of Privacy and Autonomy

The fundamental question whether it is ethically justified to intervene in people’s privacy by means of ERR technologies arises, as emotions are generally regarded as something rather personal and private, hence particularly worthy of protection (Roemmich et al., 2023). The general goal of ERR is to promote positive and counteract those which are defined as negative. As mentioned above (section “Ethical Justification of ERR in AAL”), technology developers present ERR as a tool to enhance the well-being and quality of life of users. The hypothesis behind this claim is that negative emotions have a detrimental effect on one’s health and well-being, while positive emotions are supposed to be beneficial. It is quite plausible that people, in general, prefer to be confronted with situations that make them happy rather than sad or angry. However, this does not lead to the conclusion that negative emotions should be suppressed at all costs, since dealing with them can represent an essential part of processing negative experiences in one’s life, e.g. in situations of increasing need of assistance, which AAL users may be confronted with.

From a medical perspective, the links between emotions and human health have been investigated in several contexts and studies (for a review see Smith & Weihs, 2019). Smith and Weihs (2019) stress that the quality of social relationships plays an important role in experiencing and expressing emotions. They argue that the psycho-social emotion regulation learned in the course of life is much more important to one’s health than the prevalence of positive emotions over negative ones. How we deal with our emotions seems much more important to our health than the emotions themselves. Psychological findings support this view as the suppression of negative emotions can have pathological effects, whereas the acceptance of negative emotions may yield positive health effects (Ford et al., 2018).

Due to the reductionist view of emotions that forms the basis of these systems, it may be regarded as questionable that they are actually able to interfere in an autonomy-undermining way with the user’s highly personal process of emotion regulation. However, these systems assume that there is a “hidden truth” (Crawford, 2021, p. 153) to be discovered and that machine learning algorithms are much more capable of uncovering it than humans themselves. In case of one’s private feelings this clearly states a violation of one’s right to “emotional privacy” (Roemmich et al., 2023), i.e. the right to decide to hide one’s emotions from others, especially if automatic ERR is integrated into AAL systems that a potentially vulnerable group of persons relies on to maintain a certain degree of autonomy in their daily lives.

The issue becomes even more apparent if one considers the evaluative part of emotions. As for example Nussbaum states, emotions inform us about which things are important for our well-being, flourishing and individual conception of a good life (Nussbaum, 2001). We grieve, because we have lost an important person that was an essential part of our life, or we may be upset because of our increasing dependency and need of care, as these indicate that we may be no longer able to live the life that we were used to. These are negative emotions that most people would rather prefer not to experience. However, they are legitimate expressions of what we feel and are essential parts of our individual self. If a system keeps notifying care providers about detected negative emotions as sadness or anger, this may interfere with the users’ highly personal choice to express and process their emotions in their private lives, as they may feel pressed to no longer overtly express their emotions to avoid further notifications of others.

### Human Variability and Machine Normativity

Another ethical aspect regarding machine learning techniques in general is the tendency to normalise human practices. Machine learning techniques lead to a normalisation of the data: Larger deviations from the norm get discarded to enable algorithmic calculations, and the majority label continues to be used in further processing in the case of unclear data (Mohammad, 2022). This causes the variability to be ignored that exists both among humans (e.g. their neurodiversity, Mohammad, 2022) and within an individual, and thus leads to a misinterpretation of human behaviour. Additionally, historical normalisation needs to be mentioned, as the calibration data is always data from the past, which is used as a predictor for future behaviour (Mohammad, 2022). Behavioural changes or spontaneous emotional reactions and expressions thus fall under suspicion of pathology. Normalisation of previously determined expressions of emotions can lead to the invalidation of deviant forms or expressions that are hard to read for the system: “AI systems convey to the user what is “normal”; implicitly invalidating other forms of emotion expression.” (Mohammad, 2022, p. 7). As mentioned above, normalisation in favour of positive emotions is problematic. Positive as well as negative feelings are essential parts of our personal and social self and can provide important psycho-social impulses for interacting with other humans.

## Conclusion

The present article discusses various types of ERR technology currently developed for the use in AAL systems, their theoretical basis, and the crucial ethical challenges connected to them. We argue that a reductionist view that reduces emotions to physiological elements is ethically questionable. Such a view ignores the complex interplay between different evaluative, conscious and unconscious elements. It also disregards how the socio-cultural context shapes the individual experience and regulation of emotions. The technological design in the vast majority of the analysed papers solely focuses on bodily or physiological signals. The underlying concepts of emotions (BET and CMA) are simplified to allow operationalisation, and it seems rather questionable that they are a reliable basis to capture the authentic emotional experience of a user. From an ethical perspective, we also argue that there are no clearly justifiable reasons for developing a technology that aims to track the emotional states of a vulnerable group of users, as emotions are a very sensitive and private aspect of a person. It remains unclear if and how automatic ERR can support the autonomy of users and general goals of AAL. The technological attempts to regulate emotions that are seen as negative are questionable, as these emotions are to a certain extent a legitimate expression of what we deem as essential parts of our personal lives. Hence, the majority of the ethical aspects that we discussed above would also apply to more complex theoretical and technological approaches to emotions and automatic ERR in AAL systems.

Ignoring the complexity and individuality of emotions as well as the contextual factors which shape emotional reactions undermines the very goals of AAL-technology. Narrowing down this complex issue in terms of a solutionist approach might also obscure alternatives– above all the improvement of intersectoral healthcare as well as social services– to the use of technology. It is highly questionable whether ERR technologies are the right approach to ensure the quality of life of their users and create personalised healthcare services.

### Limitations

Our paper only discusses ethical issues that are specifically related to current attempts in AAL technological development to include ERR in AAL systems. Ethical discussion and research strategies should be broadened to include all forms of assistive technologies in healthcare so that further conclusions can be drawn. Further research is needed on whether and how vulnerable groups (e.g. people suffering from dementia) could profit from an integration of ERR in AAL, which is a central aspect for ethical justifiability of this technology. This would require collaborations between different sciences as well as participatory studies with potential users that address the wishes and needs of the users.
